# AI-driven complex systems redefine cognitive science

**DOI:** 10.1016/j.xinn.2026.101314

**Published:** 2026-02-13

**Authors:** Peng Wang, Xu Sun, Liye Zou, Effie Lai-Chong Law, Fred Paas

**Affiliations:** 1 Department of Language, Literature and Communication, Faculty of Social Sciences and Humanities, Vrije Universiteit Amsterdam, Amsterdam 1081 HV, the Netherlands; 2 Department of Psychology, Education, and Child Studies, Erasmus School of Social and Behavioural Sciences, Erasmus University Rotterdam, Rotterdam 3062 PA, the Netherlands; 3 Faculty of Science and Engineering, University of Nottingham Ningbo China, Ningbo 315100, China; 4 Nottingham Ningbo China Beacons of Excellence Research and Innovation Institute, Ningbo 315100, China; 5 Body-Brain-Mind Laboratory, School of Psychology, Wuhan Sports University, Wuhan 430079, China; 6 Department of Computer Science, Durham University, Durham DH1 3LE, UK; 7 School of Education, University of New South Wales, Sydney, NSW 2052, Australia

Dear Editor,

Human behavior and cognition emerge from dynamic interactions across neural, psychological, social, and environmental levels, producing phenomena from simple actions such as picking up a dropped pen to complex processes such as learning, mood regulation, or symptom change. These processes are multi-scale and non-linear, unfolding from milliseconds (e.g., neural firing) to years (e.g., habit formation), forming interconnected, adaptive systems. In step with this reality, cognitive science is shifting toward complex systems thinking, which frames cognition as an emergent property of interacting components rather than a product of isolated causes.^1^ Concepts from complexity science—non-linear dynamical systems, attractor states, coupled networks, and critical transitions—clarify sensitivity to initial conditions and shifts between stable and unstable states, offering richer accounts than static, reductionist models. This shift is visible across domains, exemplified by psychiatry here: temporal-network and dynamical-systems approaches recast disorders as evolving configurations of mutually reinforcing symptoms that can exhibit attractors (e.g., persistent low mood), bifurcations, and critical transitions such as rapid onsets of suicidal crises.^2^ Hour-scale fluctuations in suicidal ideation underscore system instability and the need for time-sensitive, dynamic risk models.^3^ More broadly, the central question of cognitive science moves from “which factor causes outcome X?” to “how do elements interact within a complex system to generate X?”—a pivot enabled by high-dimensional, longitudinal data and methods that treat variability as signals rather than noise.^4^ Against this backdrop, advances in artificial intelligence (AI)/machine learning (ML) make it feasible to model high-dimensional, temporally embedded data and to simulate evolving trajectories at scale (Figure 1 illustrates how AI/ML technologies unify these domains to advance complex systems thinking).

**Figure 1:**
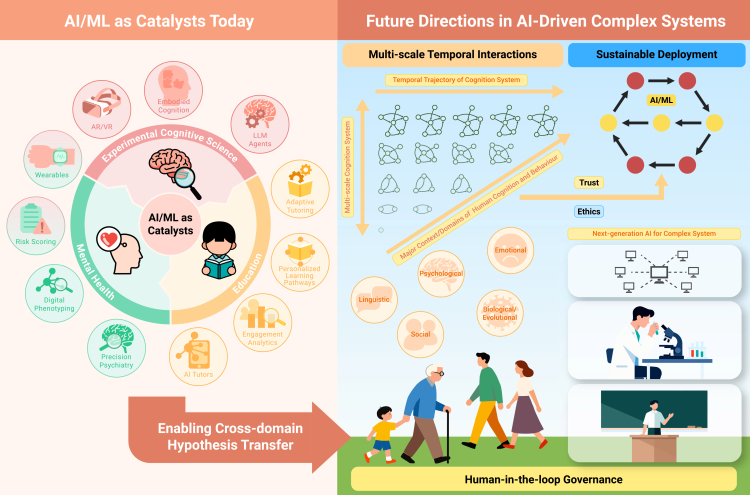
AI and complex systems thinking enabling integrative advances in cognitive science Left: overview of AI/ML technologies (e.g., LLMs, RNNs, and transformers) enabling applications in cognition, mental health, and education. Right: future directions including multi-scale modeling, trustworthy and ethical frameworks, and sustainable deployment. The arrows illustrate the synergies between cognition, mental health, and education and how future directions could integrate these fields.

Our integrating route is straightforward: we synthesize emerging developments and highlight how human cognition is increasingly modeled as an adaptive system, with AI acting either as a simulator—probing stability, controllability, and tipping points through simulated behavioral trajectories—or as an estimator—inferring latent states, couplings, and transition structures from heterogeneous, time-dependent data. In doing so, we offer a cross-domain framework—linking cognition, mental health, and education—that preserves domain-specific nuance while charting the AI-catalyzed shift from reductionism to complex dynamics.

## AI as a catalyst for a new era of complexity

Below, we explore AI’s role in three domains to demonstrate how it starts to enable cognitive science to model complex, evolving systems (see the left image in Figure 1). Throughout this discussion, “AI model” denotes any learning model—typically Deep Neural Network (DNN) based—that maps data to predictions or latent dynamics. “AI system” is the end-to-end application that embeds such models with data pipelines, interfaces, and governance. “AI agent” refers to a goal-directed component capable of planning and tool use.

### AI for broader cognition

AI agents—used as simulators of human-like reasoning—allow cognitive theories to be stress tested at scale in classic paradigms of planning, abstraction, and social inference (e.g., Tower of Hanoi/Blocks World, analogical reasoning sets, and false-belief or faux-pas benchmarks).^5^ Complementing this macro lens**,** AI models often act as a middle ground between estimator and simulator of latent decision dynamics. Tiny recurrent neural networks (RNNs; small RNNs used for modeling individual decision strategies) with 1–4 units trained per participant recover trial-level strategies in concrete tasks: in two-armed bandits, they dissociate learning-rate asymmetries for gains versus losses; in probabilistic reversal learning, they capture stickiness and sensitivity to volatility across reversals; and in intertemporal choice, they reveal state-dependent discounting after rare, large outcomes. By reading out next-choice probabilities from a low-dimensional state, these models make trial-by-trial preference updates visible and thus operate as hypothesis-generating tools rather than strict mechanistic accounts.^6^

### AI for mental health and psychology

In psychiatry, AI frequently serves as an estimator with digital phenotyping pipelines that integrate smartphones, wearables, and even web history to predict near-term mood shifts, relapse risk, and treatment response. Observable inputs include sleep regularity and duration, heart-rate variability, step counts, GPS entropy (mobility diversity), call/text rhythms, and speech features such as pause rate and prosody. Liu et al. show that wearable-derived physiological features can predict adolescent mental health risks with high accuracy, enabling early intervention.^7^ Transformer approaches such as life2vec encode life-event sequences—for example, diagnoses followed by job change and relocation—into vector representations that predict outcomes (including mortality risk) and personality facets while yielding interpretable concept embeddings.^8^ Extending this idea, LLM-based early-warning schemes can fuse daily speech snippets and activity traces, embed acoustic-text-behavioral features, estimate movement between symptom-network states (e.g., sleep disruption ↔ low mood), and trigger clinician or patient feedback when the probability of a tipping point rises.^2^

### Personalized learning and educational AI

Adaptive tutoring systems often combine the simulator and estimator roles to deliver fine-grained personalization. At the learner level, models perform error-type tagging (e.g., algebraic sign errors versus concept errors), update mastery estimates per skill via knowledge tracing, and simulate policy choices such as practice schedules and item difficulty. At the classroom level, the system treats teacher-student-content as a coupled network in which strategies and misconceptions diffuse across peers: item-level responses update skill vectors, a spaced-retrieval planner proposes next activities with uncertainty bands, and a dashboard supports teacher decisions. Although this evidence base is newer than in clinical contexts, the mechanisms already align with complex-systems theory, and agentic tutors that analyze error patterns in algebra to adapt exercises illustrate how these pipelines translate into concrete learning gains.^9^

Across psychology, mental health, and education, a common set of complex systems principles recur—despite differences in context. Feedback loops govern affect regulation in depression and learning adaptation in classrooms, emergent patterns shape both symptom dynamics and group engagement, and multi-scale interactions unfold across neural, behavioral, and environmental levels. What unites these applications is not a shared dataset or domain but a modeling paradigm: AI systems that can capture time-sensitive, interdependent processes. Together, these models do more than predict—they validate and refine cognitive theories at scale. Some unify broad domains, while others dissect individual mechanisms. By simulating behaviors beyond the limits of traditional experiments (e.g., perturbing trajectories and scaling temporal windows), AI enables a generative, systems-oriented science of cognition that bridges theory and application. Some scholars caution that AI models may serve more as analogies than mechanistic explanations, highlighting the need for careful interpretation and strong theoretical grounding.

## Challenges and future directions

### Trustworthy cognitive and educational AI: Risks, remedies, and neuro-AI synergy

Advanced AI is hard to trust in practice: reasoning is often opaque and outputs can be unreliable. Typical failures include hallucinated or ungrounded responses, overfitting on small datasets, poor sensitivity to context, performance drift across populations and sites, and biased error rates for underrepresented groups (e.g., under-detection of depression).^10^ These issues block scientific and educational deployment, where explainability and accountability are essential.

In response, we outline remedies tied to realistic deployments in cognitive science and education: first, reduce hallucination and improve factuality by grounding generative outputs in approved sources (retrieval-augmented generation for classroom content and clinical guidelines), constraining outputs with rubrics/templates, and requiring source attributions for instructor/clinician review; second, improve interpretability and accountability by pairing high-capacity models with readable surrogates (sparse/linear approximations and sensitivity maps) and calibrated uncertainty that is visible to teachers and clinicians; third, guard against drift and poor transportability using preregistered stress tests, site-specific cross-validation, and local recalibration when models move across schools, clinics, or populations; fourth, audit equity with subgroup performance reports and apply fairness-oriented training when disparities appear; and finally, keep humans in the loop; each prediction or recommendation should include a brief rationale tied to concrete evidence (e.g., symptom trajectories or curriculum-aligned exemplars), with escalation workflows for ambiguous cases. Linking model patterns back to cognitive and neuroscientific theory helps move from correlations to plausible mechanisms.

### Two-way agenda (neuro-AI)

Beyond using AI to study minds, methods from cognition and neuroscience can improve AI itself: theory-driven benchmarks (composition, hierarchy, and memory), representational comparisons with brain/behavior data, human-inspired curricula and active learning, and closed-loop experiments that shape inductive biases. This bidirectional program fits a complex-systems view and strengthens both cognitive science and AI development.

### Data ethics and governance

Privacy, security, and governance are central for high-dimensional longitudinal data from wearables, digital phenotyping, and life-sequence records. Conventional consent regimes cannot cope with modern AI’s scale, repurposability, and inferential reach. Beyond baseline anonymization and secure storage, we therefore require governance that distributes control (data trusts), aligns design with stakeholder values (participatory co-design), and makes performance auditable (model cards and bias audits). For individual control, Finland’s MyData initiatives offer an operational template for granular consent. Together, embedding these practices within cognitive-science workflows enables fairness, accountability, and trust.

### Research and implementation priorities

Future research must prioritize several interconnected directions (illustrated by the right image in Figure 1).

(1) Developing next-generation AI/ML architectures that are explicitly designed to capture multi-scale temporal interactions and emergent phenomena in cognition and that support cross-domain hypothesis transfer (e.g., applying education peer-network dynamics to psychiatric symptom-propagation models and analyzing the implications of mental health tipping-point models for the development of educational interventions targeting student burnout). Concretely, the same complex-systems toolkit underlies these domains. In each case, we represent entities (e.g., learners, cognitive strategies, or symptoms) as nodes in a dynamical network linked by directed influences, estimate these couplings from longitudinal traces (trial-by-trial decisions, digital phenotyping streams, and classroom interaction logs), and use AI models to simulate how local perturbations propagate through the system. This shared framework—feedback loops, attractor states, and early-warning indicators of tipping points—explains why educational peer-network models of strategy diffusion and psychiatric symptom-propagation models can borrow methods and hypotheses from one another.
(2) Establishing robust methodologies for validating dynamic models against long-term human data across diverse contexts, including embedding model-derived decision strategies into real systems (e.g., integrating Tiny-RNN-inferred strategies into adaptive teaching to test for measurable learning gains).
(3) Building trustworthy systems through participatory ethical frameworks, algorithmic fairness auditing, and transparent human oversight mechanisms.
(4) Enabling sustainable deployment via low-resource AI solutions, effective augmentation models (e.g., AI supporting teachers/clinicians), and cross-cultural adaptation strategies.

Addressing these challenges will determine whether this paradigm shift can fulfill its potential to advance our understanding of the mind and enhance global human well-being.

## Conclusion

This letter illustrates how AI and complex systems theory are reshaping cognitive science, moving from reductionist to holistic models. Across psychology, psychiatry, and education, AI harnesses longitudinal data to build dynamic models of cognition, thus forecasting outcomes and enabling personalized interventions. By integrating AI with complex systems thinking, cognitive science is driving practical innovations that enhance human potential and societal well-being. Interdisciplinary frameworks combining systems science, neuroscience, and computer science, alongside ethical AI deployment, will unlock new insights into learning, memory, and mental health, marking a new era of scientific discovery.

## Funding and acknowledgments

This work was supported by the 10.13039/501100004543China Scholarship Council Program (project ID: 202208060121), Shenzhen Educational Research Funding (grant no. zdzb2014), the Shenzhen Science and Technology Innovation Commission Foundation (grant no. 202307313000096), the Social Science Foundation of China’s 10.13039/501100002338Ministry of Education (grant no. 23YJA880093), the Science and Technology Innovation Projects of the State General Administration of Sports (23KJCX057), the 10.13039/501100002858China Postdoctoral Science Foundation (grant no 2022 M711174), the National Center for Mental Health Foundation (grant no. Z014), Research Excellence Scholarships of 10.13039/501100009019Shenzhen University (grant no. ZYZD2305), research funding for the Society of Sport Science (grant no PT2023030), the 10.13039/100000001National Science Foundation of Shenzhen University (grant no. 000311), the Science and Technology Innovation Projects of the State General Administration of Sports (23KJCX057), and the Guangdong Youth Health Research Fund (grant no 2024WT006). The funders had no role in the study design, data collection and analysis, decision to publish, or preparation of the manuscript.

## Declaration of interests

The authors declare no conflicts of interest.

## References

[1] Kok A.A.L., Huisman M., Giltay E.J. (2025). Adopting a complex systems approach to functional ageing: bridging the gap between gerontological theory and empirical research. Lancet Healthy Longev..

[2] Öngür D., Paulus M.P. (2025). Embracing complexity in psychiatry—from reductionistic to systems approaches. Lancet Psychiatry.

[3] Coppersmith D.D.L., Ryan O., Fortgang R.G. (2023). Mapping the timescale of suicidal thinking. Proc. Natl. Acad. Sci. USA.

[4] Scheffer M., Bockting C.L., Borsboom D. (2024). A dynamical systems view of psychiatric disorders—theory: a review. JAMA Psychiatry.

[5] Binz M., Akata E., Bethge M. (2025). A foundation model to predict and capture human cognition. Nature.

[6] Li J.-A., Benna M.K., Mattar M.G. (2025). Discovering cognitive strategies with tiny recurrent neural networks. Nature.

[7] Liu J.J., Borsari B., Li Y. (2025). Digital phenotyping from wearables using AI characterizes psychiatric disorders and identifies genetic associations. Cell.

[8] Savcisens G., Eliassi-Rad T., Hansen L.K. (2024). Using sequences of life-events to predict human lives. Nat. Comput. Sci..

[9] Yan L., Greiff S., Teuber Z. (2024). Promises and challenges of generative artificial intelligence for human learning. Nat. Hum. Behav..

[10] Obermeyer Z., Powers B., Vogeli C. (2019). Dissecting racial bias in an algorithm used to manage the health of populations. Science.

